# Gallic Acid as an Oxygen Scavenger in Bio-Based Multilayer Packaging Films

**DOI:** 10.3390/ma10050489

**Published:** 2017-05-03

**Authors:** Astrid F. Pant, Sven Sängerlaub, Kajetan Müller

**Affiliations:** 1 Chair of Food Packaging Technology, Technical University of Munich, Weihenstephaner Steig 22, 85354 Freising, Germany; 2 Fraunhofer Institute for Process Engineering and Packaging IVV, Giggenhauser Str. 35, 85354 Freising, Germany; sven.saengerlaub@ivv.fraunhofer.de (S.S.); kajetan.mueller@ivv.fraunhofer.de (K.M.); 3 Faculty of Mechanical Engineering, University of Applied Sciences, Bahnhofstraße 61, 87435 Kempten, Germany

**Keywords:** food packaging, absorber, active packaging, polyphenol

## Abstract

Oxygen scavengers are used in food packaging to protect oxygen-sensitive food products. A mixture of gallic acid (GA) and sodium carbonate was used as an oxygen scavenger (OSc) in bio-based multilayer packaging films produced in a three-step process: compounding, flat film extrusion, and lamination. We investigated the film surface color as well as oxygen absorption at different relative humidities (RHs) and temperatures, and compared the oxygen absorption of OSc powder, monolayer films, and multilayer films. The films were initially brownish-red in color but changed to greenish-black during oxygen absorption under humid conditions. We observed a maximum absorption capacity of 447 mg O_2_/g GA at 21 °C and 100% RH. The incorporation of GA into a polymer matrix reduced the rate of oxygen absorption compared to the GA powder because the polymer acted as a barrier to oxygen and water vapor diffusion. As expected, the temperature had a significant effect on the initial absorption rate of the multilayer films; the corresponding activation energy was 75.4 kJ/mol. Higher RH significantly increased the oxygen absorption rate. These results demonstrate for the first time the production and the properties of a bio-based multilayer packaging film with GA as the oxygen scavenger. Potential applications include the packaging of food products with high water activity (a_w_ > 0.86).

## 1. Introduction

In active packaging technology, oxygen (O_2_) scavengers are used to protect O_2_-sensitive food products [1,2,3]. Current examples include scavenger sachets, which are not common in Europe, and active packaging materials containing O_2_-scavenging substances in the polymer matrix [1,3]. Scavengers can also be classified according to their active substances, i.e., the substance that reacts with O_2_. Many commercially available scavengers are based on iron, oxidizable polymers, or sulfite [3,4,5,6,7].

Materials based on renewable resources support the development of sustainable packaging. Research has therefore focused on the development and improvement of bio-based packaging materials [8,9,10]. Some natural substances can be used as bio-based O_2_ scavengers including plant extracts, tocopherol, ascorbic acid, and especially polyphenols, which are known for their ability to react with O_2_ [11,12,13,14]. 

One polyphenol that is potentially suitable as an O_2_ scavenger for packaging applications is gallic acid (3,4,5-trihydroxybenzoic acid, GA) because it absorbs large amounts of O_2_ under alkaline conditions [13,15,16]. To provide alkaline conditions in the packaging material, GA must be combined with a base. Furthermore, the presence of water is required [13,16]. During the reaction between GA and O_2_, a change of color from white (pure GA) to dark brown, dark green, or black may be observed, depending on which base is used [13]. Therefore, GA was also proposed as an O_2_ indicator [13,16,17].

The incorporation of GA into packaging films has not been investigated in great detail. Langowski and Wanner first mentioned gallic acid as an O_2_ scavenger for packaging applications [16]. Goldhan et al. and Wanner described application trials, in which combinations of GA and different bases were integrated either in the adhesive layer or the coating of packaging films [13,17]. Recently, Ahn et al. reported the production of monolayer films containing GA and potassium carbonate [18]. However, the use of GA as a scavenger in packaging films requires multilayer structures comprising an outer O_2_ barrier layer to limit O_2_ ingress from the environment, and an inner food contact layer to prevent direct contact between GA and the packed food. 

In this study, we describe the O_2_ absorption properties of bio-based packaging films containing GA as a natural O_2_ scavenger. Multilayer packaging films entirely based on renewable resources were produced in a three-step process involving compounding, cast film extrusion, and lamination. We investigated the effects of the polymer matrix, the relative humidity, and the temperature on oxygen absorption and determined the change in surface color caused by the reaction. The results provide insight into the O_2_ scavenging properties of GA and its potential applications in food packaging.

## 2. Results

### 2.1. Film Properties

**Figure 1:**
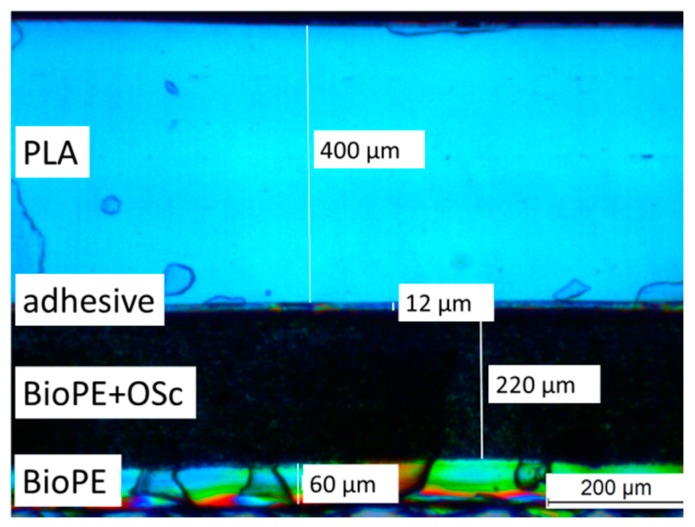
The multilayer structure of the bio-based packaging film containing the gallic acid scavenger (OSc).

A multilayer packaging film was produced in a pilot-scale three-step process involving compounding, cast film extrusion, and lamination. The film comprised a food contact layer (BioPE), an active layer containing the scavenger (BioPE + 15% (w/w) OSc), a bio-based adhesive, and an outer barrier layer (PLA) that reduces O_2_ ingress from the environment. The thicknesses of the individual layers of the produced film were determined in a microscopic analysis of the cross section and are given in Figure 1. 

The film had a brownish red color after production with some small darker particles. These particles were most probably OSc powder agglomerates formed during processing. Powder agglomerates may affect the mechanical properties and barrier properties of the film. Thus, the process must be optimized in order to avoid agglomerates.

**Figure 2:**
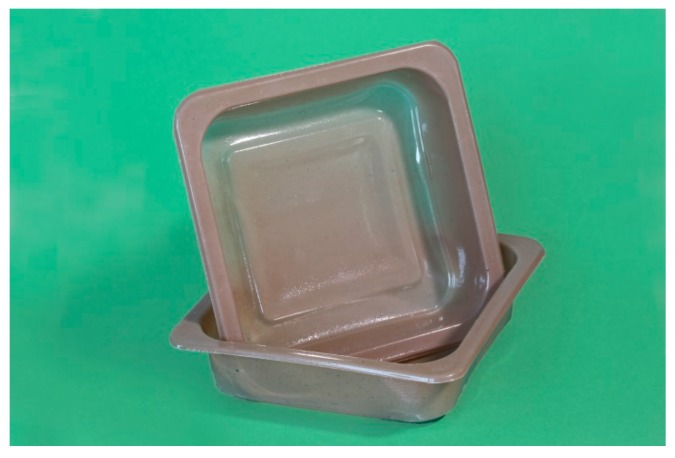
Thermoformed trays (144 mm × 144 mm × 40 mm) containing gallic acid as the O_2_ scavenger.

One potential application of the bio-based multilayer film is the production of O_2_-scavenging food packaging trays. In such a packaging setup, the main function of GA would be to scavenge residual O_2_ from the package headspace. Preliminary tests showed that the films can be thermoformed into trays (Figure 2). Future studies will therefore include storage tests with oxygen-sensitive food products to evaluate the GA-based scavenger under real packaging conditions.

### 2.2. Film Color

**Figure 3:**
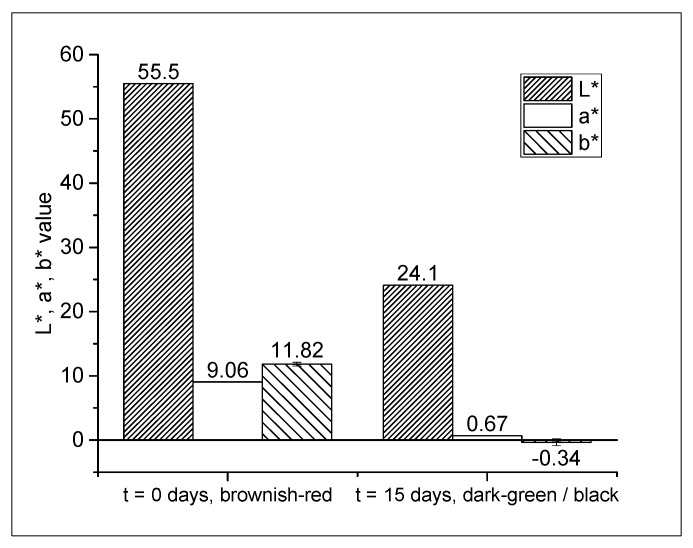
Multilayer film color after production (t = 0) and after storage at 21 °C and 100% RH (t = 15 days), expressed as CIE L*a*b* values.

The dry OSc mixture was white in color, but the first step of film production (compounding) resulted in light-purple compound pellets. This color change indicated that GA had reacted with O_2_ during the process. The multilayer film showed a darker brownish-red color, probably reflecting an additional reaction with O_2_. When stored under humid conditions, the film rapidly turned into a dark greenish-black (Figure 3), which is reflected by a decrease in L*, a*, and b* values.

Alkaline conditions promote a rapid reaction between GA and O_2_ and can be provided combining a basic substance and water [16]. This is reflected by the observed color changes during processing, which are slow under dry conditions but accelerate during storage at higher humidities. The color of polyphenols is determined by the presence of chromophoric groups that interact with light [14]. Dark reaction products of polyphenol oxidation, e.g., brown or black polycondensates, can be found in wine or humic substances [19,20]. The color change of the multilayer films indicated that GA formed larger molecules during the reaction with O_2_. Although these reaction products have not yet been characterized, they may represent dimers, as previously reported by Tulyathan et al. [15].

Remarkably, the observed color change was not proportional to the O_2_ absorption of GA. During the O_2_ absorption experiments (see Section 2.3), we noticed that the films continued absorbing O_2_ when the color had already turned into black, i.e., in the later stages of the reaction, the film color could not be related to the amount of O_2_ that was absorbed. This limits the O_2_-indicating function of GA to the first stages of the reaction. However, assessing the potential of GA as an O_2_ indicator requires further investigation of the correlation between color and O_2_ absorption.

### 2.3. Oxygen Absorption of Gallic Acid

O_2_ absorption was measured in closed cells with a defined initial O_2_ concentration of 20% (v/v) by monitoring the decrease in headspace O_2_ partial pressure over time. Varying storage conditions were used to determine the effect of temperature, relative humidity, and the polymer matrix on O_2_ absorption by GA. For better comparison, the results of the O_2_ absorption measurements are expressed as mg O_2_/g GA.

#### 2.3.1. Effect of Temperature

**Figure 4:**
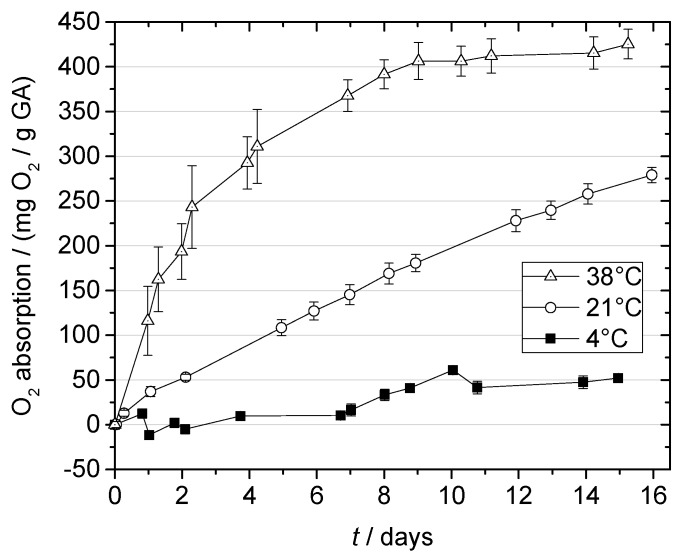
O_2_ absorption by gallic acid (GA) in multilayer films at 100% RH and varying temperatures.

The application temperature (i.e., the storage temperature of the packed product) is an important factor for the performance of O_2_ scavengers in terms of their absorption rate [7,21,22,23,24]. As described above, the O_2_ scavenging effect of GA relies on the chemical reaction of GA with oxygen. Therefore, a positive influence of temperature on the O_2_ absorption rate could be expected. We examined the O_2_ absorption by the produced multilayer films at 4, 21, and 38 °C during 16 days of storage. The results are presented in Figure 4. Higher storage temperatures led to increased O_2_ absorption; temperature had a significant effect on the initial reaction rate (*p* < 0.05).

The initial reaction rate constants *k*_init_, obtained from the initial linear increase in O_2_ absorption, were 4.2 ± 0.6, 20.0 ± 0.5, and 124.0 ± 5.8 mg O_2_/(g GA d) for 4, 21, and 38 °C, respectively. This implies that O_2_ absorption in the beginning of the measurement was twice as high at 21 °C and 5 times higher at 38 °C compared to 4 °C. 

In the linear fit of ln *k*_init_ vs. 1/*T*, Arrhenius-like behavior was observed (R^2^ > 0.99). The temperature-dependence of the initial rate constant *k*_init_ can therefore be described with the following equation:

$$k_{\mathrm{init}}\;\left(T\right)=6.90\cdot 10^{13}{\mathrm{mg}\;O}_{2}/\left(g\;\mathrm{GA}\cdot d)\;\exp(-75.4\frac{\mathrm{kJ}}{\mathrm{mol}}/\left(R\cdot T\right)\right).$$

The activation energy (*E_a_*) of O_2_ absorption by the multilayer films was 75.4 kJ/mol. Lower *E_a_* values in the range of 44.1 to 49.0 kJ/mol have been reported for commercially available iron-based scavengers [25,26]. Hence, for the analyzed films, the temperature has a greater effect on the GA-based scavenger than on the iron-based scavengers. The positive effect of temperature on the activity of the scavenger film can be explained as follows: The two main processes that determine O_2_ absorption of the scavenger film, O_2_ transport through the polymer matrix and the chemical reaction between O_2_ and GA, are both temperature-dependent and can be described by the Arrhenius law [27,28]. Therefore, the O_2_ absorption of the film is accelerated at higher temperatures.

These results reveal the influence of temperature on the performance of GA-based O_2_ scavengers and should therefore be taken into account for future applications. 

#### 2.3.2. Effect of Relative Humidity

**Figure 5:**
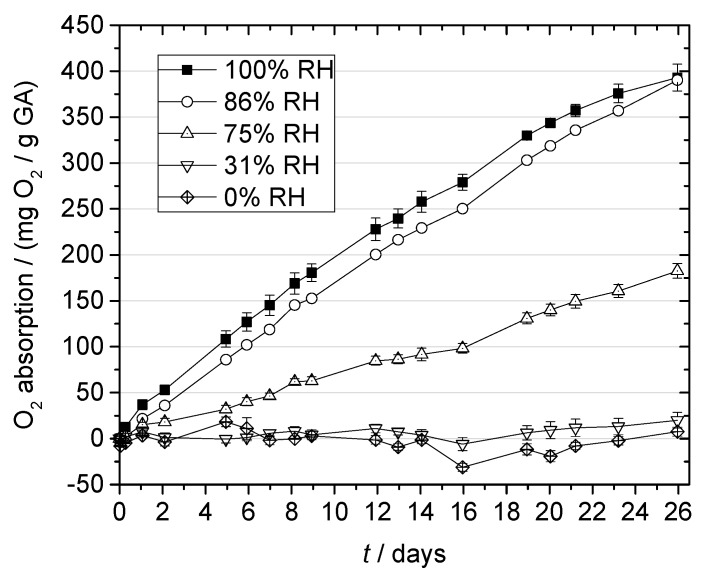
O_2_ absorption by gallic acid (GA) incorporated in multilayer films at 21 °C and different relative humidities (RHs).

Humidity, i.e., the availability of water, has been described as an important trigger of the scavenging reaction by GA [13,16,18]. The scavenging reaction is initiated by the deprotonation of GA in alkaline solution [29]. To enable this acid–base reaction in the produced multilayer films, humidity from the environment is needed. In our experiments, we determined the effect of RH on the O_2_ absorption by GA by storing multilayer films at 21 °C under different RH conditions (Figure 5). The RH conditions were chosen to simulate typical storage conditions for different types of food, characterized by different water activities (*a_w_*), i.e., dry powder products or cookies (31% RH), jam (75% RH), gammon or salami (86% RH), and beverages (100%) [22,30,31].

The amount of water present in the environment of the film significantly influenced O_2_ absorption by GA (*p* < 0.05). After 26 days, the samples stored at 100% or 86% RH achieved the highest absorption of ca. 390 mg O_2_/g GA, followed by those stored at 75% RH, which absorbed 185 mg O_2_/g GA. In comparison, storage at 31% or 0% RH resulted in negligibly low O_2_ absorption.

Remarkably, we found that the differences in O_2_ absorption were not proportional to the differences in RH. Higher RH led to a disproportionate increase in O_2_ absorption rate, whereas O_2_ absorption after 26 days was not significantly different at RH of 86% and 100% (*p* < 0.05). More research is therefore needed to characterize the influence of water on O_2_ scavenging by GA in more detail, and for quantitative analysis, a parameter other than RH may be required.

Overall, our results show that humid conditions are necessary for GA to scavenge O_2_ rapidly, and we therefore recommend GA for the packaging of moist products with water activities >0.86. 

#### 2.3.3. The Effect of Film Structure

**Figure 6:**
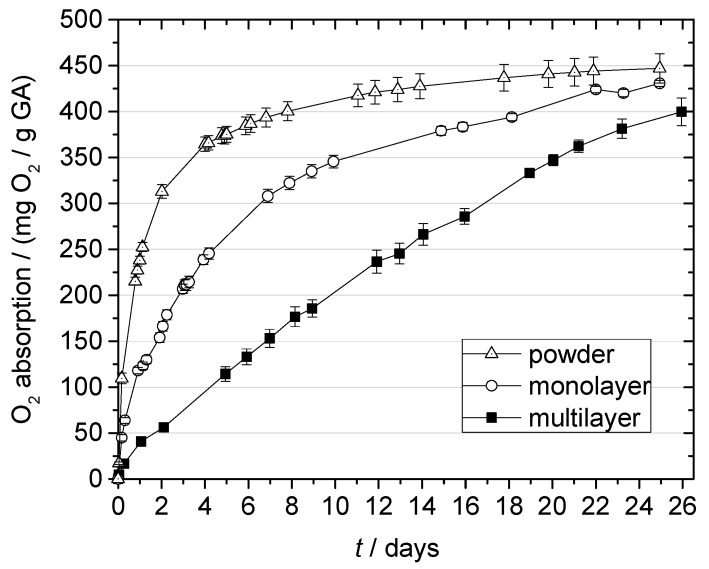
O_2_ absorption by gallic acid (GA) in mono- and multilayer films and in powder form at 21 °C and 100% RH.

The incorporation of GA into a polymer matrix led to a reduced O_2_ absorption, especially during the first few days of the reaction (Figure 6). 

After 2 days, the OSc powder absorbed twice as much O_2_ as the monolayer films. As expected, this effect was even more pronounced in multilayer films. In the powder experiment, the maximum O_2_ absorption capacity of the GA scavenger was determined to be 447 ± 16 mg O_2_/g GA, and this was achieved after 25 days storage at 21 °C and 100% RH, after which the O_2_ absorption remained constant. The measured O_2_ absorption capacity is in line with the results of Wanner who reported an absorption capacity of 340 ± 7 cm^3^ O_2_/g GA for a GA-based scavenger at 25 °C, 100% RH, and 1013 hPa (equal to 445 mg O_2_/g GA) [13]. Ahn et al. observed a much lower maximum absorption capacity of app. 64 cm^3^ O_2_/g GA for GA combined with potassium carbonate in monolayer films stored at 23 °C and 95% RH [18]. However, this difference might be attributed to different processing conditions and film structures.

Compared to commercially available O_2_ scavengers, such as iron-based or polymer-based systems, GA shows a remarkably high absorption capacity. Table 1 gives an overview of the absorption capacities of different O_2_ scavengers. For a better comparison, O_2_ absorption is related to the amount of scavenger additive used, e.g., the amount of the OSc mixture in this study.

The monolayer and multilayer films absorbed 430 or 380 mg O_2_/g GA during the 25-day storage period but did not achieve their maximum absorption capacity due to their lower absorption rate (Figure 6). However, it seems like the film production process, which involved temperatures up to 220 °C, did not considerably reduce the O_2_ absorption capacity of GA compared to the untreated OSc powder.

The slower reaction of monolayer and multilayer films may reflect the gas barrier properties of the polymer matrix. Before the reaction with GA, the O_2_ must dissolve in the polymer and diffuse to the immobilized scavenger. The same applies for water vapor, which is also necessary for the reaction. Thus, the films can be regarded as complex reaction-diffusion systems. The reaction rate of such systems is determined by both the diffusivity of the reactants (O_2_ and water vapor) in the matrix and the rate constant for the actual scavenging reaction. The current research of our group focuses on developing a mathematical model that describes O_2_ scavenging films as a reaction–diffusion system, accounting for both O_2_ and water permeation. The aim is to get a deeper understanding of the underlying reaction mechanism, thereby providing further guidance for the design of tailor-made GA-based scavengers.

## 3. Materials and Methods 

### 3.1. Materials

The scavenger (OSc) was obtained by blending gallic acid (GA) monohydrate powder (99%) and water-free sodium carbonate (Na_2_CO_3_), both from ABCR (Karlsruhe, Germany) at a ratio of 2:1. GA is a weak acid with the formula C_6_H_2_(OH)_3_COOH. At room temperature, it is a yellowish-white crystalline powder. At 253 °C, GA decomposes to form CO_2_ and pyrogallol [35].

A bio-based linear low-density polyethylene (BioPE, SLL218, Braskem, Brazil) served both as the polymer matrix for the OSc powder and as the food contact layer. BioPE was chosen due to its high O_2_ permeability (see Table 2) in order to allow for rapid oxygen transport from the packaging headspace to the embedded OSc particles.

A 400 µm polylactide (PLA) film produced at Fraunhofer IVV (Freising, Germany) from PLA IngeoTM2003 D (Natureworks LLC, Minnetonka, MN, USA), served as an outer barrier layer for the packaging film.

For lamination, the bio-based adhesive EpotalR P100 ECO was mixed with 3% (w/w) hardener Basonat LR9056 (BASF SE, Ludwigshafen, Germany). 

The thermal properties and the gas permeability of BioPE and PLA are given in Table 2.

The thermal properties were determined by differential scanning calorimetry (DSC) using a DSC 821^e^ (Mettler-Toledo, Columbus, OH, USA) under a nitrogen atmosphere. Two heating runs (23 °C to 300 °C) were made with a heating rate of 10 K/min. The degree of crystallinity *X*_c_ was calculated by relating the determined melting enthalpy ∆ *H*_m_ to the theoretical melting enthalpy of 100% crystalline samples, which was taken to be 93.7 J/g (PLA) or 277.1 J/g (LDPE), respectively [36,37].

All gas permeation measurements were carried out on two replicate samples of 50 µm films.

O_2_ permeability was determined at 23 °C and 50% RH according to the DIN 53 380 standard method using an Oxtran device (Mocon, Brooklyn Park, MN, USA).

The water vapor transmission rates were determined according to DIN EN ISO 15106-3 with a Brugger device (WDDG, Brugger, Germany) at 23 °C and an 85–0% gradient of RH. 

### 3.2. Film Production

#### 3.2.1. Compounding

The OSc powder (15% (w/w)) was melt-blended with BioPE in a co-rotating twin screw extruder (Collin Teach-Line Bench Top Compounder ZK 25 T × 24 D; Dr. Collin GmbH, Ebersberg, Germany). The melt strain was then cooled on dry ice and pelletized (Pelletizer CSG 171 T; Dr. Collin GmbH). The temperature profile of the process comprised five zones at 20/100/125/115/120 °C. The rotational screw speed was 80 rpm, the melt pressure was 125 bar, and the melt temperature at the rod die was 160 °C. The BioPE-OSc compound was stored in hermetically sealed aluminum bags under a nitrogen atmosphere at 23 °C. The final concentrations of GA, Na_2_CO_3_, and BioPE in the compound were 10%, 5%, and 85% (w/w), respectively.

#### 3.2.2. Cast Film Extrusion

Monolayer and multilayer cast films were produced on a flat film co-extrusion line (Dr. Collin GmbH) with a nozzle width of 300 mm.

Monolayer film: The BioPE-OSc compound was extruded by applying temperatures of 60/120/140/160/180/180 °C (zones 1–6) in an extruder with an L/D ratio of 30.

Multilayer film: An additional BioPE layer was co-extruded onto the monolayer film. The temperature profile in the extruder (L/D = 24) was 60/160/180/200/220 °C (Zones 1–5). The feedblock and nozzle temperatures were 200 °C and 220 °C, respectively.

#### 3.2.3. Lamination

The co-extruded film was laminated to a 400 µm PLA film (lacquering and lamination pilot plant, Fraunhofer IVV, Freising, Germany). To improve surface properties, both films were Corona-discharge-treated beforehand. The adhesive was applied using a gravure roll.

The resulting laminate was packed in an aluminum bag under a nitrogen atmosphere. It was stored for 24 h at 50 °C to ensure complete hardening of the adhesive and thereafter stored at 23 °C.

#### 3.2.4. Thermoforming

Samples of the bio-based multilayer films (ca. 650 cm^2^) were thermoformed into packaging trays using a semi-automatic thermoforming device (LDFG23B, Illig, Germany) in order to assess the potential of the film for thermoforming applications. The heating time was 12 s with a heating plate temperature of 520 °C. The moulding time was 6 s. The tray dimensions were 144 mm × 144 mm × 40 mm. In the present study, there was no further analysis of the trays.

### 3.3. Film Characterization

#### 3.3.1. Layer Thickness

Microtome cut cross sections (20 µm) were obtained using a Jung Autocut 2055 (Leica, Wetzlar, Germany). Micrographs of these cross sections were made with a transmitted light microscope (Leitz GmbH, Wetzlar, Germany) and the thicknesses of the individual layers were determined using the corresponding microscope software (LAS V 4.0, Leica, Wetzlar, Germany).

#### 3.3.2. Color Measurement

The surface color of the monolayer and multilayer films was analyzed before (t = 0) and after oxygen absorption (15 days at 21 °C and 100% RH) using the non-digital color imaging system DigiEye (DigiEye v2.62, VeriVide, UK). The system settings and calibration have been described by Böhner et al. [38]. Average surface color was determined from measurements of 10 points evenly distributed over the film surface and expressed as CIE L*a*b* values. In the CIE L*a*b color space, the L* axis gives the lightness. The a* axis represents the red/green opponent colors, and the b* axis represents the yellow/blue colors.

#### 3.3.3. Oxygen Absorption

Film samples were stored in stainless steel cells equipped with two valves for gas flushing. For a detailed description, see Rieblinger et al. [39]. The cells were hermetically closed with a glass lid and had a free headspace volume of 86 cm^3^ or 106 cm^3^.

Relative humidity in the cells was adjusted with silica gel, calcium chloride, sodium chloride, potassium chloride, or water (0, 31, 75, 86, and 100% RH at 21 °C, respectively) [40,41,42].

The initial headspace gas atmosphere of 20% O_2_ and 80% N_2_ (v/v) was established by flushing the cell with synthetic air (Linde Gas, Munich, Germany). The decrease in the headspace O_2_ partial pressure (p_O2_) was then measured non-destructively during storage using Fibox 4 Trace, a luminescence-based oxygen detection system (PreSens Precision Sensing GmbH, Regensburg, Germany). For this, an optical sensor spot (PSt3) was placed inside the cell at the glass top. 

The following samples were analyzed: 

OSc powder: 0.06 g;

Monolayer: 0.25 g film: ~2.5 × 5 cm;

Multilayer: 0.96 g film ~2.5 × 5 cm.

All O_2_ absorption measurements were carried out on at least three replicate samples of films or powder, except for the measurement at 86% RH, for which only one replicate is available.

The results (decrease in p_O2_) were converted into the mass of absorbed O_2_ (m_O2_) using the ideal gas law: m_O2_ = (p_O2_ V_HS_ M_O2_)/(R T), where V_HS_ denotes the headspace volume of the cell, M_O2_ the molar mass of O_2_, R = 8.13446 J/(mol K) the ideal gas constant, and T is the temperature in Kelvin. For better comparison, m_O2_ was normalized to the mass of the GA contained in the film or in the scavenging powder, so all O_2_ absorption values are given in mg O_2_/g GA.

The initial rate constants of O_2_ absorption *k*_init_/(mg O_2_/(g GA day)) were determined from the initial absorption values (up to 130 mg O_2_/g GA) by linear regression. In the initial phase of O_2_ absorption, the influence of the reactant concentrations on the reaction rate can be neglected so that this simplified approach of estimating *k*_init_ can be used.

The temperature dependence of *k*_init_ was analyzed by fitting the linearized form of the Arrhenius equation ln(*k*) = (−Ea/R)(1/T) + ln (*A*).

### 3.4. Data Treatment 

OriginPro 2016G (OriginLab Corp., Northampton, MA, USA) was used for the analysis of the absorption data. The effect of treatments was examined in a one-way analysis of variance (ANOVA); the null hypothesis was rejected with a significance level of 0.05.

The presented results denote the arithmetic means of at least three replicate samples; error bars represent the standard deviation.

## Figures and Tables

**Table 1:** O_2_ absorption capacities of different O_2_ scavengers.

Scavenger Type	Absorption Capacity mg O_2_/g Scavenger	References
OSc powder (gallic acid + sodium carbonate, 2:1)	298	this study
polymer additive with iron powder (SHELFPLUS^®^ O_2_ 2710)	39–48	[5]
polymer additive: copolyester-based polymer (Amosorb DFC 4020, Colormatrix Europe, Liverpool, UK)	43–47	[32]
polymer: ethylene methylacrylate cyclohexenylmethyl acrylate ‘OSP™’	60–100	[23,33]
polymer: metal-catalysed poly(1,4-butadiene)	140	[34]
coating: cyclo-olefin bonded to a silicate backbone ‘ORMOCER^®^’	90	[6]

**Table 2:** Properties of the used polymers.

	Thermal Properties			Gas Permeability	
Polymer	Glass Transition Temperature °C	Melting Temperature °C	Crystallinity Degree %	O_2_ cm^3^ (STP) 100 µm/(m^2^ d bar)	H_2_O g 100 µm/(m^2^ d)
**BioPE**	n.d.	125.6	24.6	1898	1
**PLA**	60.1	152.3	39.1	153	58
